# 

**Published:** 1900-12

**Authors:** 


### BOOKS RECEIVED.

Transactions of the Texas State Medical Association. Thirty-second annual session held at Waco, Texas, April 24–27, 1900. H. A. West, M. D., Secretary.

Report of the Commissioner of Education for the Year 1898-99. Volume I. Washington: Government Printing Office. 1900.

A Text-Book of Histology, including Microscopic Technic. By A. A. Böhm, M. D., and M. von Davidoff, M. D., of the Anatomical Institute in Munich. Edited, with extensive additions to both text and illustrations, by G. Carl Huber,

M. D., Junior Professor of Anatomy and Director of the Histological Laboratory, University of Michigan. Authorised translation from the second revised German edition, by Herbert H. Cushing, M. D., Demonstrator of Histology and Embryology, Jefferson Medical College, Philadelphia. Octavo, pp. 501, 351 illustrations. Philadelphia and London: W. B. Saunders & Co. 1900. [Price, $3.50 net.]

International Clinics. A Quarterly of Clinical Lectures and Especially Prepared Articles on Medicine, Neurology, Surgery, Therapeutics, Obstetrics, Pediatrics, Pathology, Dermatology, Diseases of the Eye, Ear, Nose and Throat, etc. Edited by Henry W. Cattell, A. M., M D. With the collaboration of John B. Murphy, M. D., of Chicago; Alexander D. Blockader, M. D., of Montreal; H. C. Wood, M. D., of Philadelphia; T. M. Rotch, M. D., of Boston; E. Landolt, M.D., of Paris; Thomas G. Morton, M. D., and Charles H. Reed, M. D., of Philadelphia. Volume III. Tenth series. 1900. Philadelphia: J. B. Lippincott Co. 1900.

A Manual of Materia Medica and Pharmacology. Comprising all organic and inorganic drugs, which are and have been official in the United States Pharmacopeia, together with important allied species and useful synthetics. For students of medicine, druggists, pharmacists and physicians. By David M. R. Culbreth, M. D., Professor of Botany, Materia Medica and Pharmacognosy in the Maryland College of Pharmacy, Baltimore. New (2d) edition. In one 8vo volume of 881 pages, with 464 illustrations. Philadelphia and New York: Lea Brothers & Co, Publishers. 1900. [Cloth, $4.50 net.]

A Manual of Hygiene and Sanitation. By Seneca Egbert, A. M., M. D., Professor of Hygiene in the Medico-Chirurgical College of Philadelphia. New (2d) and revised edition. In one handsome 12mo volume of 427 pages, with 77 engravings. Philadelphia and New York: Lea Brothers & Co., Publishers. 1900. [Cloth, $2.25 net.]

A Pocket Text-Book of Obstetrics. By David J. Evans, M. D., Lecturer on Obstetrics and Diseases of Infancy in McGill University Faculty of Medicine, Montreal. In one handsome 12mo volume of 409 pages, with 149 illustrations, partly in colors. Lea's Series of Pocket Text-Books. Edited by Bern B. Galladet, M. D. Philadelphia and New York: Lea Brothers & Co. 1900. [Cloth, $1.75 net; full flexible leather, $2.25 net.]

Sexual Debility in Man. By Frederic R. Sturgis, M. D., formerly Clinical Professor of Venereal Diseases, Medical Department, University of the City of New York; Ex-Visiting Surgeon to the City Hospital, Blackwell's Island; author of "A Manual of Venereal Diseases;" one of the authors of "A System of Legal Medicine," etc, etc. Small 8vo, pp. 432. Illustrated. New York: E. B. Treat & Co. 1900. [Price, $3.00.]

